# ProteinArchitect: Protein Evolution above the Sequence Level

**DOI:** 10.1371/journal.pone.0006176

**Published:** 2009-07-15

**Authors:** Matthias Haimel, Karin Pröll, Michael Rebhan

**Affiliations:** 1 Department of Bioinformatics, Upper Austrian University of Applied Sciences, Hagenberg, Austria; 2 Friedrich Miescher Institute for Biomedical Research, Basel, Switzerland; Monash University, Australia

## Abstract

**Background:**

While many authors have discussed models and tools for studying protein evolution at the sequence level, molecular function is usually mediated by complex, higher order features such as independently folding domains and linear motifs that are based on or embedded in a particular arrangment of features such as secondary structure elements, transmembrane domains and regions with intrinsic disorder. This ‘protein architecture’ can, in its most simplistic representation, be visualized as domain organization cartoons that can be used to compare proteins in terms of the order of their mostly globular domains.

**Methodology:**

Here, we describe a visual approach and a webserver for protein comparison that extend the domain organization cartoon concept. By developing an information-rich, compact visualization of different protein features above the sequence level, potentially related proteins can be compared at the level of propensities for secondary structure, transmembrane domains and intrinsic disorder, in addition to PFAM domains. A public Web server is available at www.proteinarchitect.net, while the code is provided at protarchitect.sourceforge.net.

**Conclusions/Significance:**

Due to recent advances in sequencing technologies we are now flooded with millions of predicted proteins that await comparative analysis. In many cases, mature tools focused on revealing hits with considerable global or local similarity to well-characterized proteins will not be able to lead us to testable hypotheses about a protein's function, or the function of a particular region. The visual comparison of different types of protein features with ProteinArchitect will be useful when assessing the relevance of similarity search hits, to discover subgroups in protein families and superfamilies, and to understand protein regions with conserved features outside globular regions. Therefore, this approach is likely to help researchers to develop testable hypotheses about a protein's function even if is somewhat distant from the more characterized proteins, by facilitating the discovery of features that are conserved above the sequence level for comparison and further experimental investigation.

## Introduction

Although there is no lack of useful computational tools for the analysis of proteins and their sequences, it can be time-consuming to put the results obtained with those tools together into a coherent picture that facilitates visual comparison of different types of protein features. Such an integrated picture is often needed when a comparison at the sequence level itself is not informative enough, for example when assessing similarity search hits that are not clearly homologous to the protein of reference, or when analyzing functionally relevant subgroups in protein families or superfamilies. In such cases, a comparison of secondary structure propensities and transmembrane region predictions can help to assess similarity at a level above the sequence, which may reveal features that are conserved, but not obvious from the alignment itself. In addition, it has been shown that many functions displayed by proteins, such as molecular interactions with partners of different shapes, or regulation of molecular interactions by post-translational modifications, require a high degree of structural flexibility in particular regions of the protein. Such regions often show a propensity for “intrinsic disorder” that can be predicted based on the sequence [1], and often contain “linear motifs” that display conservation of a few key residues [2]. Therefore, a visualization of all those different types of modules would help to compare the modular architectures of proteins at different levels of resolution, including the level between the large conserved domains and the sequence itself, which this work is particularly concerned with.

Stimulated by those challenges in protein sequence analysis, in particular for proteins with large non-globular regions, we have developed a Java-based framework and Web server that produce a visualization of different protein modules, including PFAM domains that are reliably detected with HMMs (Hidden Markov Models), transmembrane domains predicted by TMHMM, secondary structure propensities predicted by PSI-PRED, and propensities for intrinsic disorder as predicted by DISOPRED2 (see Methods). The results of those relatively reliable and informative predictions are then presented in an information-dense visualization with drill-down capabilities, to facilitate visual comparison by allowing comparison of as many proteins as possible on a computer screen. Proteins are clustered by the multiple sequence alignment program ClustalW 1.83, to bring clearly similar proteins into proximity in the visualization [3]. A benefit of combining such a clustering tree and the visualization of the various predictions is that this allows easy visual verification of the results of the clustering, as modules are usually conserved in their overall arrangement in closely related groups of proteins. Proteins that display a considerably different architecture but that are considered closely related by the clustering procedure should therefore be inspected more carefully.

By making the server and the code available publicly we hope to provide starting points for those who would like to address similar challenges, and to open up the possibility of future enhancements to this tool using the sourceforge community platform. The ProteinArchitect framework has been designed in a way that should allow its extension into a more comprehensive protein analysis platform, which would help to make in depth protein analysis more accessible to more scientists.

## Results

Guided by several protein sequence analysis cases we have developed a dedicated tool that facilitates the visual comparison of different features related to the architecture of proteins. For cases in which a single protein (in the form of a FASTA sequence) is the focus of the analysis, an input mode (“Submit one sequence”) has been developed that will automatically retrieve candidate homologs from selected eurkaryotic organisms (see Species List S1) using BLAST, then analyze those sequences with a number of algorithms related to protein architecture and present the results in a compact visualization. This results display facilitates that exclusion of spurious hits with clearly different architecture, and the discovery of conserved patterns in particular regions. For cases in which a collection of proteins has been already assembled, e.g. by careful inspection of the results of sequence similarity, domain composition or structural searches, they can be used as input directly (using the “Submit a sequence collection” mode, as a list of FASTA-formatted sequences). This will trigger an analysis and visualization of the architecture of those sequences without performing additional BLAST searches. Therefore, the “Submit one sequence” mode is useful for cases in which a first overview is needed on potential homologs with similar architecture from some of the main model organisms. The “Submit a sequence collection” mode, however, provides a much more flexible way to use ProteinArchitect, in a variety of situations that include the assessment of weak hits and the analysis of different regions in the protein.

The results display we developed in the course of this project is optimized for the visual comparison of protein features and propensities for several proteins of interest, to understand similarities and differences between them. It combines protein architecture cartoon style visualizations of conserved domains with additional details in the form of secondary structure and disorder propensities, and the arrangement of the proteins using a clustering based on sequence similarity (see below). Using this visualization, proteins with weak similarity can be compared, and regions can be discovered that display particular patterns of interest.

**Figure 1:**
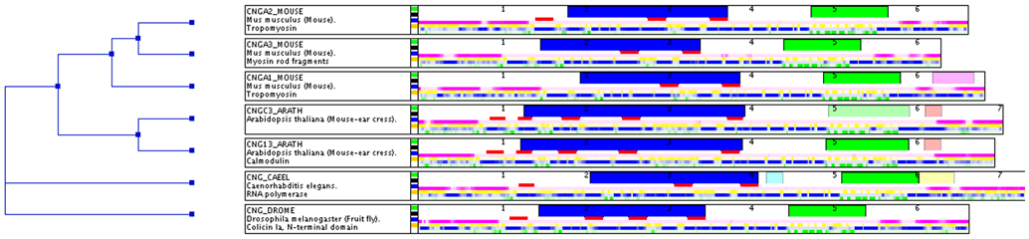
A ProteinArchitect result that illustrates several features (described in the text).

How the protein clustering is combined with the visualization of the various protein modules is illustrated in Fig. 1. The protein displayed in the center of the clustering tree (CNGC3_ARATH, ‘Probable cyclic nucleotide-gated ion channel 3’, from the plant *Arabidopsis thaliana*) contains several regions that display similarity with conserved domains described in PFAM at the Sanger Institute [4]. They are displayed as colored objects with different shapes, in this case as rectangles. The first PFAM hit shown in blue has also been found in all the other proteins in the collection. The more C-terminal PFAM hit shown in green is half transparent in this protein, while hits to the same PFAM domain in the other proteins are colored more strongly. This indicates that the level of similarity to this domain is weaker in this protein (below the gathering threshold used in the construction of PFAM families). Hovering the mouse on top of it will display additional information, such as the name of the PFAM domain, its position in the protein, the resulting similarity score and E value. This information will help to judge the relevance of the hit. Clicking on it will open a new browser window with more information on the biology of the domain and its evolutionary distribution at the PFAM server.

Comparing the different PFAM modules, it becomes apparent that all displayed proteins share the blue and the green domain, while other (weaker) hits to PFAM domains at the C-terminal end are less conserved and less significant, and therefore need to be inspected more carefully before they can be considered relevant. Below the PFAM domains a track of red rectangles is displayed, which show the positions of predicted transmembrane domains. Note that the region near the blue domain (an ion transport domain) shows putative transmembrane regions that overlap with the blue track further down, which shows propensities for alpha-helical secondary structure. As transmembrane domains often show such a tendency, this supports their prediction. However, note that the number and exact position of the transmembrane regions is not exactly the same in all proteins, which may be due to problems with transmembrane region prediction or reflect real differences. If this is of interest for understanding the proteins and their functions, this comparison would then, for example, convince the user to perform additional analysis on those regions before assuming that the location of all transmembrane regions is predicted reliably by TMHMM.

The pink track below the transmembrane region predictions reflects propensities for intrinsic disorder, while the yellow track just below it shows propensities for coil conformation. In many cases those will overlap, but in others, they differ significantly. For the proteins shown in Fig. 1, a frequently observed phenomenon is visible, namely the high degree of structural flexibility of the most N-terminal and the most C-terminal regions of the proteins. Interestingly, regions where a propensity for helix (blue tracks) or strand (green tracks) are present sometimes overlap with a tendency for intrinsic disorder. Although this may seem unexpected at first, it has been shown that some intrinsically disordered regions can harbor considerable propensities for helical or strand secondary structures. By binding a molecular partner those can be stabilized [5]. If such overlaps between intrinsic disorder and secondary structure (helix or strand) are conserved across some evolutionary distance this may indicate the presence of a functional module [6], although such regions are not well understood at present. Note also how the green PFAM domain (a cNMP-binding domain) coincides with a strong propensity for a number of beta-strands, which could indicate a globular structure stabilized by beta-sheets.

In contrast to many other bioinformatics tools, which try to predict the boundaries of structural elements, in ProteinArchitect secondary structure and disorder propensities are encoded by the relative strength of the color, to reflect the fact that the prediction of the exact boundaries of those modules is often difficult. Instead, visual comparison of feature conservation across homologs with ProteinArchitect can help to assess such cases in a more unbiased manner.

**Figure 2:**
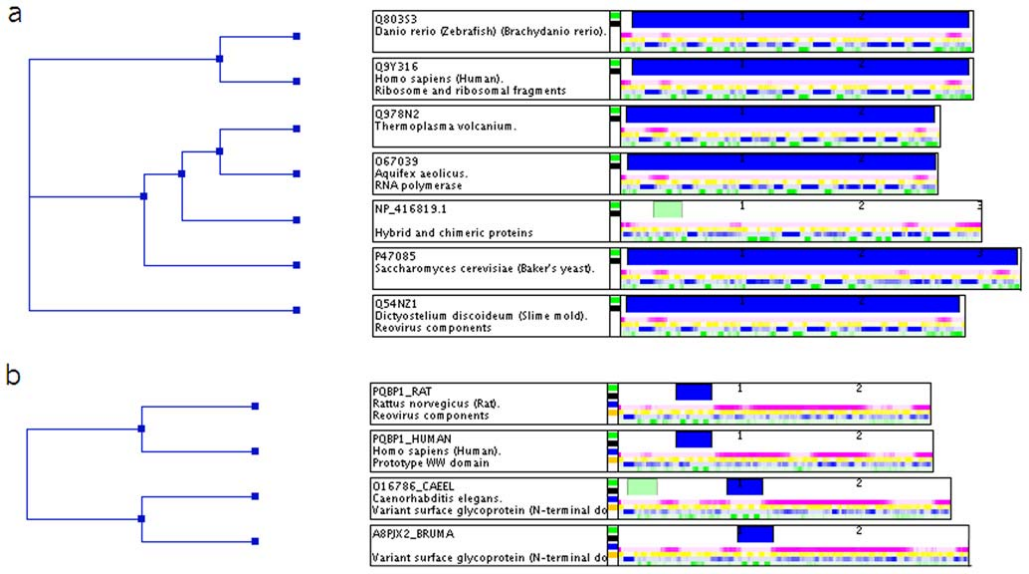
Results of similarity searches assessed with ProteinArchitect. a) The best BLAST hits in a number of proteomes are displayed. While there is a clear similarity between the eukaryotic and most prokaryotic proteins in terms of secondary structure propensities, the third protein, the best hit in E.coli, differs considerably in architecture. Therefore, this protein is not likely to be a homolog with similar structural features. b) Assessment of the results of a BLAST search in Uniprot with the “Putative uncharacterized protein” O16786_CAEEL as a query. Despite the weak similarity in most parts of the protein with mammalian proteins (31% identity), overall architecture is quite similar. To distinguish conserved from more variable features, a close ortholog of the human protein, the rat protein, has been included, as well as another nematode protein (A8PIX2_BRUMA) from Brugia malayi, which is 48% identical to the C.elegans query protein. Note also the conserved C-terminal region that shows a characteristic combination of features but no disorder prediction (pink), flanked by disordered regions with a propensity for alpha-helices (blue).

In two additional examples (Fig. 2), an assessment of the results of similarity searches using such a fine-grained architecture-based approach can be seen. In Fig. 2a, a single protein was used as input for ProteinArchitect (the human protein). In this mode (“Submit one sequence”), ProteinArchitect will do a BLAST search in several model organisms (with a focus on eukaryotes) and report the best hit for each, assuming that the number of proteins to be reported per species has not been changed in “Optional parameters”. From those results, the most informative proteins have been selected manually and displayed (using the checkboxes and “Display” button on the results page). The result is shown in Fig. 2. Using this visualization, the E.coli hit (fifth protein, NP_416819.1, ‘acetyl-CoA carboxylase, beta (carboxyltranferase) subunit’) is not a convincing homolog, while the other prokaryotic proteins from Aquifex and Thermoplasma are clearly more similar in their overall architecture (helix and strand propensities, in blue and green, and disorder, in pink). As an assessment of similarity search hits only by E value, bit score or alignment inspection can be challenging in the twilight zone [7], such a visualization can often help to sort out difficult cases. In particular, it will often allow the exclusion of spurious hits. Using known homologs with a low degree of sequence similarity, the user can become familiar with the biological meaning of different aspects of the visualization, including the conservation of features in disordered regions, which often evolve more rapidly than globular regions.

The second example for assessing the results of similarity searches in Fig. 2b demonstrates how unknown nematode proteins that do not show clear similarity in most parts of the protein to mammalian similarity search hits can display a highly similar global architecture in ProteinArchitect. Here, two groups of proteins, i.e. two mammalian proteins that are clearly globally similar to each other, and two nematode proteins that are clearly globally similar to each other based on alignment inspection, are being compared. Although many regions of the nematode proteins do not align well with their mammalian hits, propensities for disorder and secondary structure are quite similar overall in those areas as well. This can be interpreted as additional evidence beyond the alignment itself, making it more likely that the mammalian hits are indeed homologs. In addition, it facilitates the discovery of regions such as the C-terminal area that displays weak secondary structure propensities but no disorder propensity, and disordered flanks that have a propensity for alpha-helical conformation. Note that such patterns are similar to the ones described by Fuxreiter et al. [2] for regions that contain functional linear motifs.

In addition to the above usage examples, many other analysis questions can be answered using ProteinArchitect. For example, you can collect FASTA sequences from other Web servers for a particular superfamily and organisms you are interested in, and submit them for analysis and visualization using the “Submit a sequence collection” button. As this will trigger a number of different, time-consuming analyses that are needed in the background to build up the above visualizations, an email address has to be provided. Once the analysis is complete, a notice will be sent to this email address with a link to the results page. On the results page, different options are provided to fine-tune the results, select sequences of interest, drill-down into proteins or organisms of interest, and to export the data in different formats. To help with publishing and communication, a legend can be generated using a button on the results display page, and parameters used can be inspected by using the “Parameters” button.

## Discussion

Due to recent advances in sequencing technologies we are increasingly flooded with millions of predicted proteins that await comparative analysis. In many cases, mature tools focused on revealing hits with considerable global or local similarity to well-characterized proteins will be able to lead us to testable hypotheses about a protein's function, or the function of a particular region. In many other cases, however, this paradigm has its limitations, due to the absence of well-characterized proteins with sufficient similarity, or due to our lack of understanding of the function of many nonglobular regions.

One of the most useful resources in this area is the PFAM database [8], which organizes information about conserved domains in proteins and their biological relevance. For proteins of interest, their ‘architecture’ can be displayed in form of a simple cartoon that shows the relative positions of PFAM domains in the sequence. For example, CNGC3_ARATH is represented there as a protein with a large PFAM domain, plus extensive N-terminal and C-terminal sequence that does not contain known any PFAM domains, and that is therefore completely featureless in such a representation. Obtaining more granular information about the architecture is difficult and time-consuming, also at other public resources related to protein analysis.

The visual comparison of different types of protein features with ProteinArchitect will be useful when assessing the relevance of similarity search hits, as the visualization allows a comparison of proteins above the sequence level, and therefore the discovery of conserved local features that may not be obvious in the alignment itself. Also, it can be used to discover subgroups in protein families and superfamilies with similar architectures, and to understand protein regions with conserved features outside globular regions that may coincide with some propensity for conserved secondary structure [6]. Therefore, this approach is likely to help researchers to develop testable hypotheses about a protein's function even if is somewhat distant from the more characterized proteins, by facilitating the discovery of features that are conserved above the sequence level for comparison and further experimental investigation.

With ProteinArchitect, regions in the protein can be understood at greater detail. This can help to make informed decisions on the boundaries of regions that can be excised in constructs, to investigate the role of different regions of the protein. It can also help to put other information about localized properties of a protein into context. Note that, in contrast to classic bioinformatics tools, we did not attempt to predict an exact boundary of any feature. As an alternative, we used a visual representation of the strength of any prediction at a particular position in the sequence, to avoid errors, and to reflect the fact that domains and regions often do not have exact boundaries. We hope that this can help to make more informed decisions about potentially interesting regions for further investigation.

## Methods

**Figure 3:**
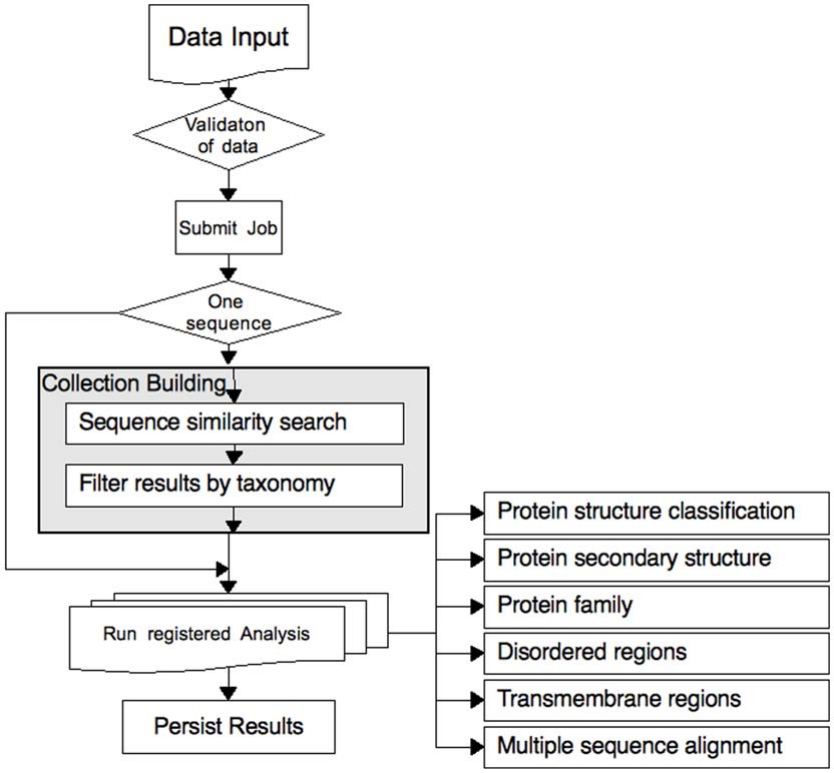
overview of the analysis flow in ProteinArchitect.

**Figure 4:**
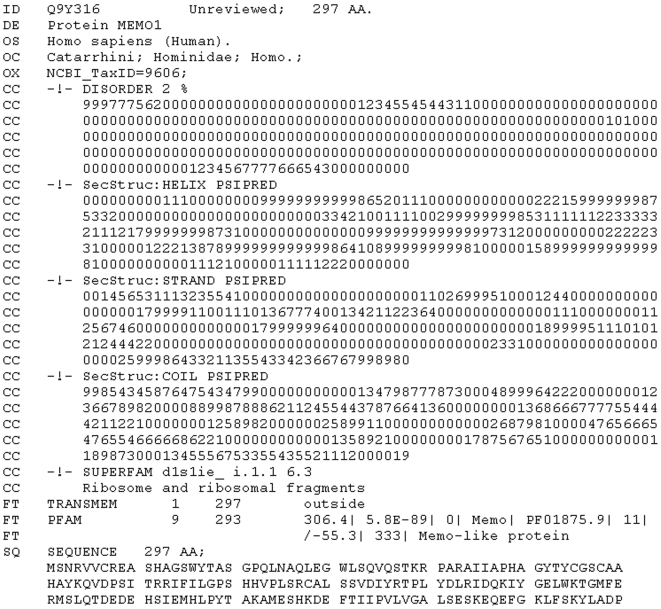
An example for the Swissprot-like format used for storing the results of the analyses shown in Fig. 3.

An overview of the analyses performed in the current version of ProteinArchitect is provided in Fig. 3. If a single protein was submitted, similar sequences are gathered from a trimmed-down database of UniProt (version 14.3, [9]) protein sequences using a BLAST search (version 2.2.10, [10]). This database only contains sequences from a selected number of organisms (see Species List S1). After filtering the resulting collection of protein sequences using the user-provided or default BLAST bit score, the protein with the highest bit score in a particular species gets selected for further analysis. The user can also select a number higher than 1 in the options to retrieve more than one protein per species. The chosen proteins are analysed as a group for clustering and as an individual to predict Pfam domains (version 23, [8]) using HMMer (version 2.3.2, [11]), Transmembrane regions using TMHMM (version 2.0, [12]), disordered regions using DISOPRED (version 2, [13]), and secondary structure propensities using PSIPRED (version 2.5, [14]). If a protein collection was submitted, the BLAST search and filtering is skipped, but all the other analyses described above are performed. Once all analyses have finished, SWISSPROT format like flat files are generated for each protein, see Fig. 4 for an example. Based on those, visualization is generated based on Java technology. The visualization of the architecture was developed, and those tracks chosen, to enable a compact, rich and simple view to compare architectures of as many proteins as possible with each other. To visualize the ClustalW (version 1.83, [3]) clustering tree (the guide tree that is based on distances calculated from pairwise alignments of all input sequences), a Java based library called ATV [15] was used. Details on the above methods, and related Java code, are available at protarchitect.sourceforge.net.

The ProteinArchitect framework was developed in an extensible manner to allow an asynchronous processing of analysis. The pipeline incorporates the concept of Design Patterns and the fragmentation of analysis into ‘smaller’ slices which increases the flexibility for the current pipeline and should facilitate further development by the sourceforge community.

ProteinArchitect makes use of DAO (Data Acccess Object), which provides functionality to store and retrieve information with specific operations. The persistence mechanism is backed by a MySQL Database server to support the needed functionality. The modular nature within a pipeline of analysis is also reflected in the software design, where one analysis of a sequence or of a sequence collection can be changed independently from the rest. By making use of the Factory method design pattern, with the ability of dynamic loading of pipeline configurations, ProteinArchitect supports the flexibility to modify, add or remove one protein analysis or the whole analysis pipeline. Each analysis feeds the results into a common model which is then processed, stored and used for the ProteinArchitect representation. Because each ProteinAnalysis module can be run independent from others, ProteinArchitect incorporates the functionality of parallel execution into the software design. This enables to extend ProteinArchitect for parallel execution on one machine with multi core CPUs or on a cluster with multiple nodes.

WEB Presentation: The Web application framework Struts is used for the Web presentation layer, which follows the MVC(Model-View-Controller) Design Pattern. The control of the Web interface (Controller) is separated from the code to load and create the results into the ProteinArchitect graphic (Model) and the HTML representation of the final output (View).

The ProteinArchitect graphic and description map is created on the fly, which uses the Composite Pattern to allow a group of objects to be treated in the same way as a single instance of an object. An easy to extend and modifiable symbol and color code schema is provided to allow customization of the ProteinArchitect graphic. ProteinArchitect uses a unique color and symbol code within one graphical result to represent the same protein features in an identical way between proteins.

## Supporting Information

Species List S1 List of species used in the ‘Submit one sequence’ mode
(0.03 MB XLS)
